# Pancreatic stone protein for early mortality prediction in COVID-19 patients

**DOI:** 10.1186/s13054-021-03704-4

**Published:** 2021-07-29

**Authors:** Mathias Van Singer, Thomas Brahier, Marie-Josée Brochu Vez, Hélène Gerhard Donnet, Olivier Hugli, Noémie Boillat-Blanco

**Affiliations:** 1 Infectious Diseases Service, University Hospital of Lausanne, Lausanne, Switzerland; 2 Department of Medicine, Neuchâtel Hospital Network, Neuchâtel, Switzerland; 3 Emergency Department, University Hospital of Lausanne, Lausanne, Switzerland

Coronavirus disease 2019 (COVID-19) pandemic is straining health care systems since December 2019 [1]. Tools to identify patients at risk of adverse outcome could optimize resource allocation.

Pancreatic stone protein (PSP) is a novel biomarker for infection and sepsis with promising results in various clinical settings [2]. A meta-analysis showed that PSP performed better than C-reactive protein (CRP) and procalcitonin for detecting infection among hospitalized patients, and that the combination of PSP and CRP further enhanced its accuracy [3]. Recently, serial measurement of PSP in patients admitted to the intensive care unit (ICU) allowed early detection of sepsis [4]. In a small case series, PSP daily monitoring was suggested as a marker of sepsis in critically ill COVID-19 patients [5].

In this prospective cohort study of COVID-19 patients in the emergency department (ED) of a teaching hospital in Switzerland, we assessed the accuracy of bedside clinical severity scores (Quick Sepsis-related Organ Failure Assessment (qSOFA) and CRB-65), PSP and CRP, which is associated with severity and mortality in COVID-19 [6], at clinical presentation for 7-day mortality and separately, ICU admission. Consecutive patients (≥ 18 years old) with symptoms of acute lower respiratory tract infection, were prospectively included in case of reverse-transcription PCR-confirmed COVID-19.

PSP was retrospectively measured in − 80° stored plasma collected in the ED (nanofluidic point-of-care immunoassay; abioSCOPE®, Abionic SA, Epalinges, Switzerland). CRP plasma concentration was determined upon admission via routine testing (immunoturbidimetrics determination; Cobas 8000 platform; Roche Diagnostics, Basel, Switzerland).

The predictive accuracy of clinical scores and host biomarkers was defined by the area under the receiver-operating characteristic curve (AUROC). Optimal cut-offs for sensitivity and specificity were determined using the Youden index. The combinatorial models were compared using the DeLong method.

All analyses were performed with STATA (version 15.0, Stata Corp, College Station, TX, USA) and R Core Team (2021). The Ethics Committee of the Vaud canton approved the study (CER-VD 2019-02283) and all patients gave their written informed consent.

Of the 173 patients included, 12 (6.9%) died (7 had limitations of life-sustaining treatment precluding ICU admission) and 37 (21.6%) were admitted to the ICU by day 7 (Table 1).The median time to death was 2.0 days (IQR 1.0, 3.5). The predicting accuracy of CRB-65 (AUROC 0.87; 95% CI 0.79–0.95), CRP (AUROC 0.83; 0.79–0.93) and PSP (AUROC 0.83; 0.74–0.92) for 7-day mortality were excellent and did not differ significantly, while the performance of qSOFA was lower compared to CRB-65 (*p* = 0.002; Fig. 1a). Figure 1b shows their optimal cut-offs for sensitivity and specificity, which had an excellent negative predictive value and a poor positive predictive value.

**Table 1:** Characteristics of study participants at inclusion in the emergency department according to 7-day mortality

Patients characteristics	Survival (*n* = 161; 93%)	Death (*n* = 12; 7%)	*p* value
Sex: female, n (%)	102 (63.4)	5 (41.7)	0.236
Age (y), years [IQR]	64.0 [52.0, 75.0]	81.50 [70.3, 83.3]	0.001
Any comorbidities, *n* (%)	110 (68.3)	11 (91.7)	0.169
Hypertension, *n* (%)	73 (45.3)	8 (66.7)	0.259
Diabetes, *n* (%)	39 (24.2)	5 (41.7)	0.320
Obesity, *n* (%)	18 (11.7)	1 ( 9.1)	1.000
Cardiovascular disease, *n* (%)	20 (12.4)	6 (50.0)	0.002
Neurologic disease, *n* (%)	14 ( 8.7)	5 (41.7)	0.002
Symptoms duration, days [IQR]	7 [4, 10]	4 [3, 8]	0.140
Respiratory rate, r/min [IQR]	24 [20, 28]	34 [25, 40.00]	0.001
Heart rate median, b/min[IQR]	85 [77, 96]	96 [90, 103]	0.034
Systolic blood pressure, mmHg [IQR]	133 [122, 144]	126 [115, 145]	0.459
qSOFA ≥ 2, *n* (%)	3 (1.9)	3 (25.0)	< 0.001
CRB-65 ≥ 2, *n* (%)	18 (11.2)	9 (75.0)	< 0.001
CRP (mg/l), [IQR]	75.0 [31.0, 140.0]	205.5 [147, 254.8]	< 0.001
PSP (ng/ml), [IQR]	70.0 [48.0, 104.0]	141.0 [98.8, 224.0]	< 0.001
Outpatient management, *n* (%)	38 (23.6)	0 (0)	0.071
7-day intermediate care unit admission, *n* (%) *	12 (7.5)	3 (25)	0.072
7-day intensive care unit admission, *n* (%)	33 (20.5)	4 (33.3)	0.295

Quick Sepsis-related Organ Failure Assessment (qSOFA): 1 point each for systolic hypotension (≤ 100 mm Hg), tachypnea (≥ 22/min), or altered mentation (Glasgow Coma Scale score ≤ 14); CRB-65: 1 point each for Confusion (Glasgow Coma Scale score ≤ 14), elevated Respiratory rate (≥ 30/min), low Blood pressure (systolic < 90 mm Hg or diastolic ≤ 60 mm Hg), age 65 years or more. *CRP* C-reactive protein; *PSP* pancreatic stone protein. *IQR* interquartile range

^*^Not including patients who went to the intermediate and the intensive care units within 7 days of inclusion

**Fig. 1:**
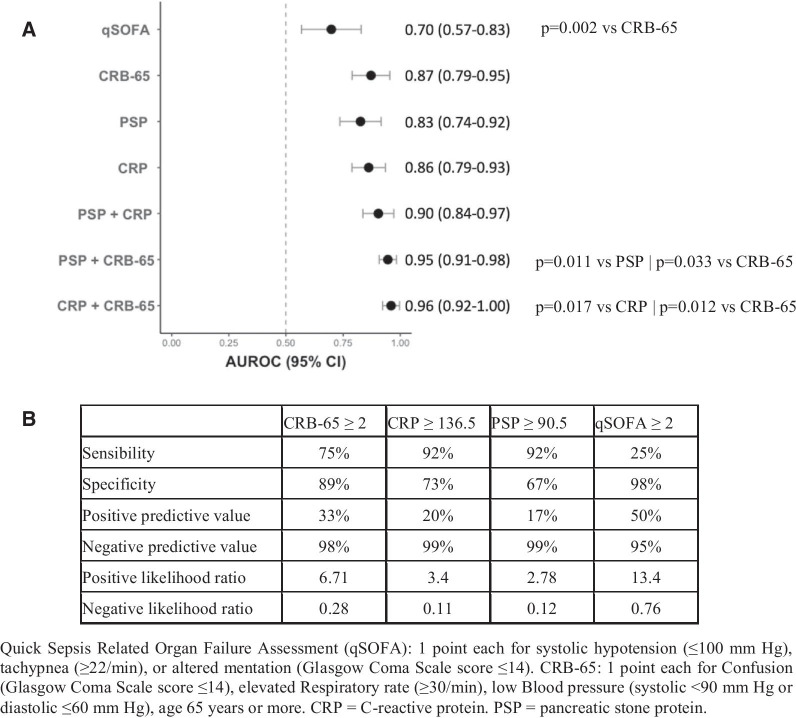
Accuracy and performance of biomarkers and clinical scores in patients with COVID-19 for 7-day mortality. **A** Nonparametric ROC curves were generated and AUROCs were plotted to illustrate the ability of bedside clinical scores and biomarkers to discriminate for 7-day mortality. **B** Sensitivity and specificity for optimal cut-offs determined using the Youden index, as well as positive predictive value, negative predictive value, positive likelihood ratio and negative likelihood ratio for the bedside clinical scores and the biomarkers are also reported

The combination of CRB-65 with biomarkers performed better than the clinical score or biomarkers alone: (1) CRB-65 plus PSP: AUROC 0.95; 0.91–0.98; *p* = 0.011 versus PSP; *p* = 0.033 versus CRB-65; (2) CRB-65 plus CRP: AUROC 0.96; 0.92–1.00; *p* = 0.017 versus CRP; *p* = 0.012 versus CRB-65 (Fig. 1a). Combination of PSP and CRP did not perform better than biomarkers or clinical scores alone.

CRP predicted 7-day ICU admission better than PSP (AUROC 0.74; 0.66–0.83 versus; 0.51; 0.41–0.61; *p* < 0.001).

The main limitations of our study are its monocentric design and the small number of patient meeting primary outcome.

CRB-65, CRP and PSP in the ED have an excellent accuracy to rule out early mortality in COVID-19. Combining CRB-65 and either biomarker improved their prognostic accuracy. As reported for sepsis, PSP appears to be a good biomarker to exclude short term risk of death [2], but not to exclude ICU admission in the context of COVID-19, suggesting different pathophysiological pathways for end-organ damage. Further research is needed to determine the clinical significance of PSP in the context of COVID-19 and its potential role as triage tool.

## Data Availability

The datasets used and analyzed during the current study are available from the corresponding author on reasonable request.

## Abbreviations

COVID-19: Coronavirus disease 2019
PSP: Pancreatic stone protein
CRP: C-reactive protein
ICU: Intensive care unit
qSOFA: Quick Sequential Organ Failure Assessment
AUROC: Area under the receiver-operating characteristic curve
IQR: Interquartile range

## References

[1] Emanuel EJ, Persad G, Upshur R (2020). Fair allocation of scarce medical resources in the time of Covid-19. N Engl J Med.

[2] Eggimann P, Que Y-A, Rebeaud F (2019). Measurement of pancreatic stone protein in the identification and management of sepsis. Biomark Med.

[3] Prazak J, Irincheeva I, Llewelyn MJ (2021). Accuracy of pancreatic stone protein for the diagnosis of infection in hospitalized adults: a systematic review and individual patient level meta-analysis. Crit Care.

[4] Pugin J, Daix T, Pagani J-L, et al. Serial measurement of pancreatic stone protein for the early detection of sepsis in intensive care unit patients: a prospective multicentric study. Critical Care. 2021;25(1):151. 10.1186/s13054-021-03576-8 PMC8056692 33879189

[5] Potential role of Pancreatic Stone Protein (PSP) as early marker of bacterial infection in COVID-19 patients [Internet]. [cited 2021 Jun 18];Available from: https://npselearning.it/img/38-vicenza/pdf/36.pdf.

[6] Kermali M, Khalsa RK, Pillai K, Ismail Z, Harky A (2020). The role of biomarkers in diagnosis of COVID-19—a systematic review. Life Sci.

